# Three-Dimensional Human Skin Models to Understand *Staphylococcus aureus* Skin Colonization and Infection

**DOI:** 10.3389/fimmu.2014.00041

**Published:** 2014-02-06

**Authors:** Lauren Popov, Joanna Kovalski, Guido Grandi, Fabio Bagnoli, Manuel R. Amieva

**Affiliations:** ^1^Microbiology and Immunology, Stanford University School of Medicine, Stanford, CA, USA; ^2^Program in Epithelial Biology, Stanford University School of Medicine, Stanford, CA, USA; ^3^Novartis Vaccines, Siena, Italy; ^4^Pediatrics, Stanford University School of Medicine, Stanford, CA, USA

**Keywords:** *Staphylococcus aureus*, MRSA, skin, colonization, organ culture

## Abstract

*Staphylococcus aureus* is both a major bacterial pathogen as well as a common member of the human skin microbiota. Due to its widespread prevalence as an asymptomatic skin colonizer and its importance as a source of skin and soft tissue infections, an improved understanding of how *S. aureus* attaches to, grows within, and breaches the stratified layers of the epidermis is of critical importance. Three-dimensional organotypic human skin culture models are informative and tractable experimental systems for future investigations of the interactions between *S. aureus* and the multi-faceted skin tissue. We propose that *S. aureus* virulence factors, primarily appreciated for their role in pathogenesis of invasive infections, play alternative roles in promoting asymptomatic bacterial growth within the skin. Experimental manipulations of these cultures will provide insight into the many poorly understood molecular interactions occurring at the interface between *S. aureus* and stratified human skin tissue.

*Staphylococcus aureus* is simultaneously a pathogen of significant morbidity and mortality and a common member of the skin microbiota. As a major human bacterial pathogen, *S. aureus* infections cause tens of thousands of deaths and millions of outpatient and emergency room visits in the United States annually (1). Although the disease caused by *S. aureus* infections manifests in a wide range of clinical presentations, the vast majority are skin and soft tissue infections (2, 3).

However, in spite of the pathogenesis associated with *S. aureus*, it is also acknowledged to be a typical member of the complex community of microbes living on and within our skin (4, 5). An estimated 20% of American adults are persistently colonized in the anterior nares with *S. aureus*, and an additional 30% are intermittently carrying *S. aureus* in the nares (6, 7). For neonates and children, the prevalence of *S. aureus* positive skin cultures is thought to be even higher (8, 9). Furthermore, evidence from recent prevalence studies in which non-nasal skin sites are surveyed suggests that traditional screening approaches may be underestimating a larger skin colonization burden of *S. aureus* by focusing on one niche, the anterior nares (10–12). Little is known about the transition from the asymptomatic colonization state to an invasive infection, despite the fact that persistently colonized individuals have nearly triple the risk of developing *S. aureus* bacteremia and are most often infected by their own colonizing strains (13–16).

Whether one considers *S. aureus* as an invasive pathogen or from the perspective of its role as a common colonizer, an appreciation of how *S. aureus* interacts with the skin environment is central to its biology. Much remains unknown about how *S. aureus* persists in the skin over time and what bacterial factors it may use to actively modify the host skin environment to persist in its replicative niche. We propose that three-dimensional (3D) human skin culture models are an informative and tractable experimental system for future investigations of the interactions between *S. aureus* and the multi-faceted skin tissue. In this perspective, we consider the current experimental model systems for studying the *S. aureus* skin interface, and describe advantages of utilizing organotypic 3D human skin models for future investigations into *S. aureus* biology.

Historically, many of the molecular biology studies into *S. aureus* skin interactions have been done using *in vitro* infections of keratinocytes cultured as a two-dimensional (2D) monolayer of cells. Such studies have provided insight into many aspects of bacterial attachment and the innate immune response mounted by keratinocytes upon encountering bacteria (17, 18). However, the bi-dimensional nature of these assays completely eludes the complex stratification and terminal differentiation process central to how keratinocytes form a multilayered epidermal tissue with barrier functionality. To study determinants of *S. aureus* interaction with a stratified skin tissue (as opposed to isolated keratinocytes), investigators have relied on a variety of animal models. Epicutaneous or superficial epidermal inoculation of *S. aureus* in rodent skin models have proven ineffective for reproducible observations of *S. aureus* growth on skin over extended periods of time (19–22). Further, there are considerable differences between human skin and rodent skin in both histology and immunology, which complicate interpretation of data with respect to bacterial localization and replication in the skin. A recently developed murine model of *S. aureus* skin and soft tissue infections uses small allergy test needles coated in bacteria to introduce the inoculum precisely and superficially into the outer ear pinna (23). This model has been used to examine the immune response to a superficial *S. aureus* skin infection (23). With this notable exception, nearly all of the existing *S. aureus* animal skin models require severe mechanical disruption of the skin to facilitate bacterial growth, such as the subcutaneous foot pad model, the scalpel wound model, or the subcutaneous skin abscess model (24–27). While these models are extremely useful for studying pathogenesis, they neither facilitate observation of the replicative niche of *S. aureus* within an intact tissue, nor address how the transition of *S. aureus* from an asymptomatic colonization state to a more invasive soft tissue or systemic infection might occur.

Due to the need for a physiologically relevant *in vitro* model system to dissect the interactions of *S. aureus* with intact human skin, we have modified an existing 3D organotypic human skin tissue model to examine the processes of staphylococcal skin colonization and infection (28, 29). An appreciation of the inherent limitations of 2D cultures for understanding skin biology has motivated the development of many tools to study stratified human skin tissue over the past several decades (30–32). Only recently, microbiologists have begun to capitalize on these advancements and utilize 3D organotypic human skin models to examine the specific interactions between the human skin and clinically relevant viral, bacterial, and fungal species (31, 33–36).

Established models for studying stratified human skin fall into two main categories, namely *ex vivo* human skin explant cultures and regenerated 3D organotypic models derived from primary cells and/or human cell lines. All 3D skin models are relatively labor and time intensive when compared to traditional 2D skin models using keratinocyte-derived cell lines. *Ex vivo* human skin explants, typically acquired from neonatal foreskin, surgical, or cadaveric tissues, can be maintained in cell culture media directly or on supports in an air–liquid interface and remain viable in culture for up to 2 weeks. Skin explants have the advantage of containing all resident cell types of the epidermis and dermis as well as skin appendages; however, there are limited options for experimental manipulation of host genetics as well as restricted availability of such tissue samples. Organotypic 3D skin models (sometimes referred to as reconstructed skin models) are generally comprised of primary or immortalized human keratinocytes, grown at an air–liquid interface on an extracellular support matrix, which can be seeded with fibroblasts (32, 37, 38). Other relevant cell types have been incorporated into organotypic skin models including melanocytes, Langerhans cells, as well as endothelial and nervous cells [reviewed in Ref. (30)]. Organotypic 3D stratified human skin cultures comprised of immortalized human cell lines such as the widely studied HaCaT cell line do not reflect the intrinsic genetic variability of cultures cultivated using primary keratinocytes, leading some to argue that models built using the former cells are more reproducible (32). On the other hand, HaCaT 3D organotypic cultures exhibit differentiation and stratification deficiencies when compared to primary keratinocyte 3D organotypic skin cultures (39–41).

In the 3D organotypic human epidermal tissue model we have modified to study *S. aureus* colonization and infection, primary human keratinocytes and fibroblasts are isolated from fresh discarded neonatal foreskin specimens (28, 29). Fibroblasts are seeded into pieces of devitalized human dermal tissue derived from cadaveric donors to provide the underlying support matrix. Keratinocytes are then seeded on top of the fibroblast-populated dermis and grow at the air–liquid interface (Figure 1B). After several days of growth, the keratinocytes fully differentiate, generating a basement membrane and all of the stratified epidermal layers, including the outermost squames of the stratum corneum (Figure 1A). The resulting 3D human organotypic tissues are composed entirely of human protein and cells, and unlike murine skin these human organotypic tissues recapitulate the thickness and most of the cellular architecture of the human epidermis and underlying dermis. A limitation of this model is the high genetic variability of skin cultures due to the use of primary cells from heterogeneous donors. Another feature of the model that needs to be improved in the future is the absence of cell types other than keratinocytes and fibroblasts, and skin appendages such as hair follicles and apocrine and eccrine sweat glands.

**Figure 1:**
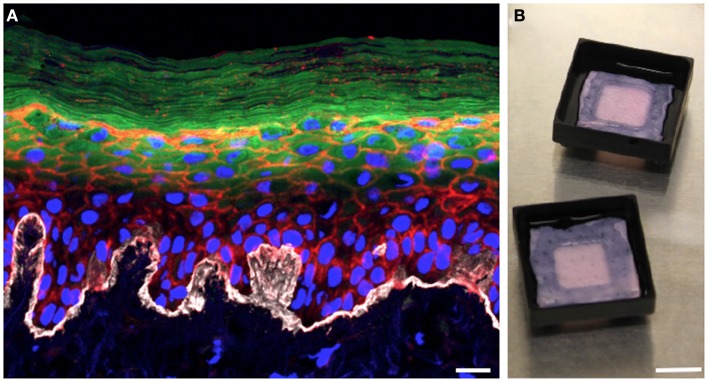
**Three-dimensional organotypic human epidermal tissues recapitulate the stratified structure of the epidermis**. **(A)** Cross-sectional view of a fully differentiated 3D human organotypic epidermal tissue. Collagen VII (white) comprising the basement membrane is visible at the interface between the dermis and epidermis. Nuclei of keratinocytes and fibroblasts are blue, filamentous actin is red, and loricrin (green) localizes to the granular layer and the squames of the stratum corneum. Scale bar is 10 μm. **(B)** Top-down macroscopic view of the 3D organotypic human epidermal tissue system. Pieces of devitalized human dermis are seeded with primary human keratinocytes and fibroblasts. Tissue culture support trays raise the organotypic human epidermal tissue and promote keratinocyte differentiation by facilitating growth at the air–liquid interface. Scale bar is 1 cm.

Epicutaneous infections of these 3D human skin cultures with *S. aureus* USA300 constitutively expressing GFP allow us to follow the bacteria during the colonization process over time. Overnight colonies of bacterial cultures grown on agar plates are re-suspended in Hanks buffer, and the inoculum is applied to the air interface of the skin culture using a pipette tip. At various times after infection, the skin cultures are harvested into paraformaldehyde fixative and further processed for cryosectioning. Using confocal microscopy to generate 3D images of infected skin tissue, we can examine the bacterial skin interface both from a “top-down” view (Figures 2B,D) as well as by looking at cross-sectional slices (Figure 2C). We have tested whether the bacteria are capable of growing on the regenerated human epidermis without exogenous addition of nutrients or media. By starting with a very small inoculum so that mainly single bacteria adhere to the corneocyte surface, we can follow the fate of the microbes over time and determine whether they grow and their preferential replication niche. By 2 days post-infection, the bacteria have expanded to form microcolonies localized at different levels within the stratum corneum of the epidermis (Figure 2, compare Figures 2A,B to later stages of growth in Figures 2C,D). Experiments done with antibiotics added to the basal media reveal this bacterial growth depends on direct interactions with the keratinocytes and not from contact with the cell culture media in the basolateral compartment of the skin cultures (data not shown).

**Figure 2:**
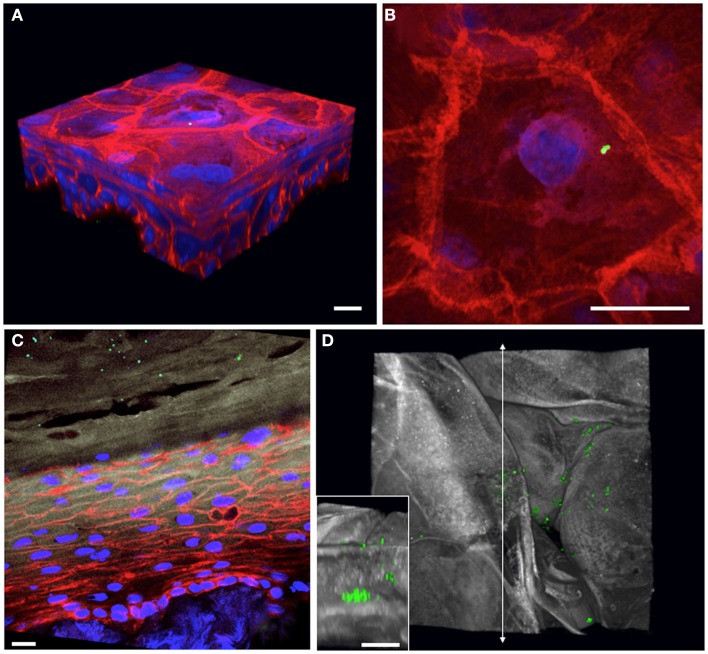
***Staphylococcus aureus* adherence, growth, and localization can be observed within 3D organotypic human epidermal tissues**. **(A)** 3D confocal microscopy reconstruction of an infected (*T* = 1 h) 5-day-old 3D organotypic human epidermal tissue. *S. aureus* USA300 GFP-expressing bacteria (in green) are visible attached to the surface squames; filamentous actin is red, and nuclei are blue. Scale bar in all panels corresponds to 10 μm. **(B)** Top-down view of bacterial inoculum in **(A)**, visualized immediately after infection. **(C)** Cross-sectional view of an 8-day-old 3D organotypic human epidermal tissue, infected with *S. aureus* USA300 GFP for 2 days. Bacteria (in green) are visible throughout the squames in the stratum corneum (loricrin in white). Filamentous actin is red and nuclei are in blue. **(D)** After 2 days of growth on the organotypic epidermal tissue, *S. aureus* USA300 GFP has started to form visible microcolonies within the squames in the stratum corneum in this top-down view (loricrin in white). Inset depicts a cross-sectional view located along the vertical line with arrows revealing a large staphylococcal microcolony.

This model system will allow us to query multiple poorly understood aspects of the interactions between *S. aureus* and skin tissue. For example, we can more precisely identify the replicative niche of *S. aureus* within the skin. Does a deeper, quiescent reservoir population of bacteria seed a more rapidly expanding population of surface associated bacteria in the stratum corneum? In addition to examining the location of bacterial attachment and growth during colonization, we can use this experimental system to observe a more pathogenic bacterial invasion of the epidermis. By modulating the maturity of the skin cultures at the time of infection, we can create conditions where bacteria are interacting with nucleated keratinocytes localized to more basal layers of the epidermis.

Unlike human or porcine skin explant models, a key advantage of an *in vitro* 3D epidermal organotypic system is that it allows the study of skin colonization and infection processes by experimentally controlling both the skin tissue as well as the bacteria (42–44). In this model, the contribution of specific host keratinocyte factors to generate an effective epidermal barrier or to provide critical innate immune responses can be examined. Human keratinocytes treated with RNAi against a gene of interest prior to seeding the dermis generate skin tissue, which is stably knocked-down for a particular gene of interest (28, 29). By then infecting these “custom knocked-down” skin tissues, in the future we can examine how tissues deficient in a particular structural or innate immune product may alter the replicative niche of *S. aureus*. Specifically, this approach could prove useful to assess the role of the host protein filaggrin in staphylococcal infections of skin cultures (45). A better understanding of staphylococcal interactions with filaggrin-depleted skin is relevant due to the contribution of filaggrin to the pathology of atopic dermatitis, a dermatological condition, which is known to enhance *S. aureus* skin colonization as well as pre-dispose patients to staphylococcal skin infections (46–48). Indeed, 3D organotypic human skin cultures depleted for filaggrin using siRNA technology have been reported in the dermatological literature (49).

In addition to experimentally manipulating the host side of the system, we can query the impact of specific bacterial virulence factors in promoting growth and invasion processes. The role played by *S. aureus* virulence determinants in skin persistence is an area ripe for inquiry. A handful of staphylococcal virulence factors have known host targets within the skin, most notably the exfoliative toxins, which cleave epidermal desmogleins, leading to staphylococcal scalded skin syndrome (50, 51). However, the vast majority of *S. aureus* virulence factors have been investigated solely for their role in contributing to invasive disease and remain largely ignored from the perspective of asymptomatic colonization. Considering the evolutionary pressure on *S. aureus* for effective transmission and the relatively infrequent occurrence of invasive infections relative to the high prevalence of colonization, it is likely that many staphylococcal virulence factors are involved in establishing and maintaining the colonization state. Clumping factor B is a clear example of a *S. aureus* virulence factor with dual roles in both the pathogenesis of a systematic infection and colonization. In bloodstream infections, clumping factor B binds to the α-chain of fibrinogen, promoting platelet aggregation, and staphylococcal attachment to damaged tissue in endocarditis (52, 53). Nevertheless, it is one of the few known critical determinants of nasal colonization and has been shown to facilitate adhesion to the keratinized epithelium through its interaction with cytokeratin 10 expressed on squamous cells (18, 54). Similarly, the secreted toxin α-hemolysin has been shown to promote the cleavage of E-cadherin in epithelia such as the lung and the skin in disease models (55). It seems very likely that the ability of *S. aureus* to modify host adherens junction biology may contribute to its ability to persist in the skin over time in a disease-free state. The 3D organotypic human epidermal tissue system will facilitate examination of the vast arsenal of *S. aureus* virulence factors with respect to the hypothesis that virulence factors may play unique roles in the biology of *S. aureus* colonization. One can assess the importance of a particular bacterial product through competition experiments where the same epidermal tissue is co-infected with wild type (WT) and isogenic mutant *S. aureus* strains. Each strain can be tracked via unique fluorophores, allowing observation of the fate of WT and mutant populations within the same stratified human tissue over time.

Examining the immune response to *S. aureus* cutaneous colonization and infections is an important potential application of a 3D human skin culture system. The bi-dimensional nature of traditional *S. aureus* infections of immune cell cultures *in vitro* forces direct interactions between the bacteria and immune cells, which are not likely to represent the most physiologically relevant way in which an immune cell would encounter *S. aureus*. By incorporating relevant immune cell types such as Langerhans cells or neutrophils into the 3D skin culture system, future investigations could examine how *S. aureus* and immune cells interact with one another in stratified human epidermal tissue. Other investigators have successfully incorporated “Langerhans-like” immune cells into similar 3D dermatological skin cultures, although to our knowledge, these models have not yet been infected for use in microbiology studies (56–58). Additionally, treatment with antibodies directed against *S. aureus* or addition of recombinant cytokines in the basolateral media of the skin cultures could allow examining impact of immune response to *S. aureus* in the localized cutaneous setting. As more *S. aureus* vaccines move forward into clinical trials, it remains totally unknown how an efficacious antibody response to vaccination may impact *S. aureus* colonization burden (59). Studies using a 3D organotypic human epidermal tissue model will prove valuable in assessing how immunization against *S. aureus* might alter bacterial population behavior on the epidermis or protect against invasive epidermal infections.

In order to better understand staphylococcal colonization and the transition to invasive infections, we must study *S. aureus* in its natural habitat: the human stratified squamous keratinized epithelium. Our group and others have begun to use organotypic 3D human skin equivalent cultures to experimentally probe *S. aureus* colonization and infections (36, 60). It is our hope that moving toward more physiologically relevant model systems, which capture the complex biology of the skin, will allow us to better understand the critical interface between our most important tissue barrier and *S. aureus*.

## Conflict of Interest Statement

Fabio Bagnoli and Guido Grandi are employees of Novartis Vaccines and Diagnostics.

## References

[1] McCaig LF McDonald LC Mandal S Jernigan DB *Staphylococcus aureus*-associated skin and soft tissue infections in ambulatory care. Emerg Infect Dis (2006) 12:1715–23 10.3201/eid1211.060190 17283622 PMC3372331

[2] Moran GJ Amii RN Abrahamian FM Talan DAD Methicillin-resistant *Staphylococcus aureus* in community-acquired skin infections. Emerg Infect Dis (2005) 11:928–30 10.3201/eid1106.040641 15963289 PMC3367577

[3] Eady EA Cove JHJ Staphylococcal resistance revisited: community-acquired methicillin resistant *Staphylococcus aureus* – an emerging problem for the management of skin and soft tissue infections. Curr Opin Infect Dis (2003) 16:103–24 10.1097/00001432-200304000-00007 12734443

[4] Grice EA Segre JAJ The skin microbiome. Nat Rev Microbiol (2011) 9:244–53 10.1038/nrmicro2537 21407241 PMC3535073

[5] Kloos WE Musselwhite MS Distribution and persistence of *Staphylococcus* and *Micrococcus* species and other aerobic bacteria on human skin. Appl Environ Microbiol (1975) 30:381–95 810086 10.1128/am.30.3.381-395.1975 PMC187193

[6] Kluytmans JJ van Belkum AA Verbrugh HH Nasal carriage of *Staphylococcus aureus*: epidemiology, underlying mechanisms, and associated risks. Clin Microbiol Rev (1997) 10:505–20 9227864 10.1128/cmr.10.3.505 PMC172932

[7] Eriksen NH Espersen FF Rosdahl VT Jensen KK Carriage of *Staphylococcus aureus* among 104 healthy persons during a 19-month period. Bull Entomol Res (1995) 115:51–60 7641838 10.1017/s0950268800058118 PMC2271555

[8] Noble WC Valkenburg HA Wolters CHC Carriage of *Staphylococcus aureus* in random samples of a normal population. J Hyg (Lond) (1967) 65:567–73 10.1017/S002217240004609X 5235259 PMC2130396

[9] Peacock SJ Justice AA Griffiths DD de Silva GD Kantzanou MN Crook DD Determinants of acquisition and carriage of *Staphylococcus aureus* in infancy. J Clin Microbiol (2003) 41:5718–25 10.1128/JCM.41.12.5718-5725.2003 14662966 PMC308978

[10] Schechter-Perkins EM Mitchell PM Murray KA Rubin-Smith JE Weir S Gupta K Prevalence and predictors of nasal and extranasal staphylococcal colonization in patients presenting to the emergency department. Ann Emerg Med (2011) 57:492–9 10.1016/j.annemergmed.2010.11.024 21239081

[11] Yang ES Tan JJ Eells SS Rieg GG Tagudar GG Miller LGL Body site colonization in patients with community-associated methicillin-resistant *Staphylococcus aureus* and other types of *S. aureus* skin infections. Clin Microbiol Infect (2010) 16:425–31 10.1111/j.1469-0691.2009.02836.x 19689469

[12] McKinnell JA Huang SS Eells SJ Cui E Quantifying the impact of extranasal testing of body sites for methicillin-resistant *Staphylococcus aureus* colonization at the time of hospital or intensive care unit admission. Infect Control Hosp Epidemiol (2013) 34(2):161–70 10.1086/669095 23295562 PMC3894230

[13] Wertheim HF Vos MC Ott AA van Belkum AA Voss AA Kluytmans JA Risk and outcome of nosocomial *Staphylococcus aureus* bacteraemia in nasal carriers versus non-carriers. Lancet (2004) 364:3–3 10.1016/S0140-6736(04)16897-9 15325835

[14] Wertheim HF Melles DC Vos MC van Leeuwen W van Belkum A Verbrugh HA The role of nasal carriage in *Staphylococcus aureus* infections. Lancet Infect Dis (2005) 5:751–62 10.1016/S1473-3099(05)70295-4 16310147

[15] Smyth DS Kafer JM Wasserman GA Velickovic LL Mathema BB Holzman RS Nasal carriage as a source of Agr-defective *Staphylococcus aureus* bacteremia. J Infect Dis (2012) 206:1168–77 10.1093/infdis/jis483 22859823 PMC3448967

[16] von Eiff CC Becker KK Machka KK Stammer HH Peters GG Nasal carriage as a source of *Staphylococcus aureus* bacteremia. Study Group. N Engl J Med (2001) 344:11–6 10.1056/NEJM200101043440102 11136954

[17] Krishna S Miller LS Host-pathogen interactions between the skin and *Staphylococcus aureus*. Curr Opin Microbiol (2012) 15:28–35 10.1016/j.mib.2011.11.003 22137885 PMC3265682

[18] O’Brien LM Walsh EJ Massey RC Peacock SJ Foster TJT *Staphylococcus aureus* clumping factor B (ClfB) promotes adherence to human type I cytokeratin 10: implications for nasal colonization. Cell Microbiol (2002) 4:759–70 10.1046/j.1462-5822.2002.00231.x 12427098

[19] Onunkwo CC Hahn BL Sohnle PG Clearance of experimental cutaneous *Staphylococcus aureus* infections in mice. Arch Dermatol Res (2010) 302(5):375–82 10.1007/s00403-010-1030-y 20130894 PMC2877165

[20] Kanzaki H Morishita Y Akiyama H Arata J Adhesion of *Staphylococcus aureus* to horny layer: role of fibrinogen. J Dermatol Sci (1996) 12:132–9 10.1016/0923-1811(95)00472-6 8814545

[21] Abe Y Akiyama H Arata J Furuncle-like lesions in mouse experimental skin infections with *Staphylococcus aureus*. J Dermatol (1993) 20:198–202 8315108 10.1111/j.1346-8138.1993.tb03861.x

[22] Kugelberg E Norström T Petersen TK Duvold T Andersson DI Hughes D Establishment of a superficial skin infection model in mice by using *Staphylococcus aureus* and *Streptococcus pyogenes*. Antimicrob Agents Chemother (2005) 49:3435–41 10.1128/AAC.49.8.3435-3441.2005 16048958 PMC1196267

[23] Prabhakara R Foreman O De Pascalis R Lee GM Plaut RD Kim SY An epicutaneous model of community-acquired *Staphylococcus aureus* skin infections. Infect Immun (2013) 81(4):1306–15 10.1128/IAI.01304-12 23381997 PMC3639601

[24] Nippe N Varga G Holzinger D Löffler B Subcutaneous infection with *S. aureus* in mice reveals association of resistance with influx of neutrophils and Th2 response. J Invest Dermatol (2010) 131(1):125–32 10.1038/jid.2010.282 20882039

[25] Cho JS Zussman J Donegan NP Ramos RI Garcia NC Uslan DZ Noninvasive in vivo imaging to evaluate immune responses and antimicrobial therapy against *Staphylococcus aureus* and USA300 MRSA skin infections. J Invest Dermatol (2011) 131:907–15 10.1038/jid.2010.417 21191403 PMC3059384

[26] Kobayashi SD Malachowa N Whitney AR Braughton KR Gardner DJ Long D Comparative analysis of USA300 virulence determinants in a rabbit model of skin and soft tissue infection. J Infect Dis (2011) 204:937–41 10.1093/infdis/jir441 21849291 PMC3156927

[27] Inoshima I Inoshima N Wilke GA Powers ME Frank KM Wang Y A *Staphylococcus aureus* pore-forming toxin subverts the activity of ADAM10 to cause lethal infection in mice. Nat Med (2011) 17:1310–4 10.1038/nm.2451 21926978 PMC3192248

[28] Ridky TW Chow JM Wong DJ Khavari PA Invasive three-dimensional organotypic neoplasia from multiple normal human epithelia. Nat Med (2010) 16:1450–5 10.1038/nm.2265 21102459 PMC3586217

[29] Kretz MM Siprashvili ZZ Chu CC Webster DE Zehnder AA Qu KK Control of somatic tissue differentiation by the long non-coding RNA TINCR. Nature (2013) 493:231–5 10.1038/nature11661 23201690 PMC3674581

[30] Auxenfans C Fradette J Lequeux C Germain L Kinikoglu B Bechetoille N Evolution of three dimensional skin equivalent models reconstructed in vitro by tissue engineering. Eur J Dermatol (2009) 19:107–13 10.1684/ejd.2008.0573 19106039

[31] Groeber F Holeiter M Hampel M Hinderer S Schenke-Layland K Skin tissue engineering – in vivo and in vitro applications. Adv Drug Deliv Rev (2011) 63:352–66 10.1016/j.addr.2011.01.005 21241756

[32] Lebonvallet N Jeanmaire C Danoux L Sibille P Pauly G Misery L The evolution and use of skin explants: potential and limitations for dermatological research. Eur J Dermatol (2010) 20:671–84 10.1684/ejd.2010.1054 20822970

[33] de Breij A Haisma EM Rietveld M Ghalbzouri El A van den Broek PJ Dijkshoorn L Three-dimensional human skin equivalent as a tool to study *Acinetobacter baumannii* colonization. Antimicrob Agents Chemother (2012) 56:2459–64 10.1128/AAC.05975-11 22290957 PMC3346587

[34] Hogk I Rupp S Burger-Kentischer A 3D-tissue model for herpes simplex virus-1 infections. Methods Mol Biol (2013) 1064:239–51 10.1007/978-1-62703-601-6_17 23996262

[35] Schaller M Schackert C Korting HC Januschke E Hube B Invasion of *Candida albicans* correlates with expression of secreted aspartic proteinases during experimental infection of human epidermis. J Invest Dermatol (2000) 114:712–7 10.1046/j.1523-1747.2000.00935.x 10733678

[36] Soong G Chun J Parker D Prince A *Staphylococcus aureus* activation of caspase 1/calpain signaling mediates invasion through human keratinocytes. J Infect Dis (2012) 205:1571–9 10.1093/infdis/jis244 22457275 PMC3415815

[37] Gangatirkar P Paquet-Fifield S Li A Rossi R Kaur P Establishment of 3D organotypic cultures using human neonatal epidermal cells. Nat Protoc (2007) 2:178–86 10.1038/nprot.2006.448 17401352

[38] Stark H-J Szabowski A Fusenig NE Maas-Szabowski N Organotypic cocultures as skin equivalents: a complex and sophisticated in vitro system. Biol Proced Online (2004) 6:55–60 10.1251/bpo72 15103399 PMC389904

[39] Boelsma E Verhoeven MC Ponec M Reconstruction of a human skin equivalent using a spontaneously transformed keratinocyte cell line (HaCaT). J Invest Dermatol (1999) 112:489–98 10.1046/j.1523-1747.1999.00545.x 10201534

[40] Maas-Szabowski N Epidermal tissue regeneration and stromal interaction in HaCaT cells is initiated by TGF. J Cell Sci (2003) 116:2937–48 10.1242/jcs.00474 12771184

[41] Schoop VM Mirancea N Fusenig NE Epidermal organization and differentiation of HaCaT keratinocytes in organotypic coculture with human dermal fibroblasts. J Invest Dermatol (1999) 112:343–53 10.1046/j.1523-1747.1999.00524.x 10084313

[42] Holland DB Bojar RA Farrar MD Holland KT Differential innate immune responses of a living skin equivalent model colonized by *Staphylococcus epidermidis* or *Staphylococcus aureus*. FEMS Microbiol Lett (2009) 290:149–55 10.1111/j.1574-6968.2008.01402.x 19054079

[43] Corriveau M-N Zhang N Holtappels G Van Roy N Bachert C Detection of *Staphylococcus aureus* in nasal tissue with peptide nucleic acid-fluorescence in situ hybridization. Am J Rhinol Allergy (2009) 23:461–5 10.2500/ajra.2009.23.3367 19807976

[44] Ohnemus U Kohrmeyer K Houdek P Rohde H Wladykowski E Vidal S Regulation of epidermal tight-junctions (TJ) during infection with exfoliative toxin-negative *Staphylococcus* strains. J Invest Dermatol (2008) 128:906–16 10.1038/sj.jid.5701070 17914452

[45] McGrath JA Uitto J The filaggrin story: novel insights into skin-barrier function and disease. Trends Mol Med (2008) 14:8–8 10.1016/j.molmed.2007.10.006 18068483

[46] Hauser CC Wuethrich BB Matter LL Wilhelm JA Sonnabend WW Schopfer KK *Staphylococcus aureus* skin colonization in atopic dermatitis patients. Dermatologica (1985) 170:35–9 10.1159/000249514 3972149

[47] Leung DY Bieber T Atopic dermatitis. Lancet (2003) 361:10–10 10.1016/S0140-6736(03)12193-9 12531593

[48] Leyden JJ Marples RR Kligman AMA *Staphylococcus aureus* in the lesions of atopic dermatitis. Br J Dermatol (1974) 90:525–30 10.1111/j.1365-2133.1974.tb06447.x 4601016

[49] Mildner M Jin J Eckhart L Kezic S Gruber F Barresi C Knockdown of filaggrin impairs diffusion barrier function and increases UV sensitivity in a human skin model. J Invest Dermatol (2010) 130:2286–94 10.1038/jid.2010.115 20445547

[50] Amagai MM Stanley JRJ Desmoglein as a target in skin disease and beyond. J Invest Dermatol (2012) 132:776–84 10.1038/jid.2011.390 22189787 PMC3279627

[51] Bukowski MM Wladyka BB Dubin GG Exfoliative toxins of *Staphylococcus aureus*. Toxins (Basel) (2010) 2:1148–65 10.3390/toxins2051148 22069631 PMC3153237

[52] Ganesh VK Barbu EM Deivanayagam CC Le BB Anderson AS Matsuka YV Structural and biochemical characterization of *Staphylococcus aureus* clumping factor B/ligand interactions. J Biol Chem (2011) 286:25963–72 10.1074/jbc.M110.217414 21543319 PMC3138276

[53] Miajlovic HH Loughman AA Brennan MM Cox DD Foster TJT Both complement- and fibrinogen-dependent mechanisms contribute to platelet aggregation mediated by *Staphylococcus aureus* clumping factor B. Infect Immun (2007) 75:3335–43 10.1128/IAI.01993-06 17438032 PMC1932920

[54] Walsh EJ O’Brien LM Liang XX Hook MM Foster TJT Clumping factor B, a fibrinogen-binding MSCRAMM (microbial surface components recognizing adhesive matrix molecules) adhesin of *Staphylococcus aureus*, also binds to the tail region of type I cytokeratin 10. J Biol Chem (2004) 279:50691–9 10.1074/jbc.M408713200 15385531

[55] Wilke GA Wardenburg JB Role of a disintegrin and metalloprotease 10 in *Staphylococcus aureus* alpha-hemolysin-mediated cellular injury. Proc Natl Acad Sci U S A (2010) 107:13473–8 10.1073/pnas.1001815107 20624979 PMC2922128

[56] Ouwehand K Spiekstra SW Waaijman T Scheper RJ de Gruijl TD Gibbs S Technical advance: Langerhans cells derived from a human cell line in a full-thickness skin equivalent undergo allergen-induced maturation and migration. J Leukoc Biol (2011) 90:1027–33 10.1189/jlb.0610374 21697260

[57] Dezutter-Dambuyant C Black A Bechetoille N Bouez C Maréchal S Auxenfans C Evolutive skin reconstructions: from the dermal collagen-glycosaminoglycan-chitosane substrate to an immunocompetent reconstructed skin. Biomed Mater Eng (2006) 16:S85–94 16823116

[58] Laubach V Zöller N Rossberg M Görg K Kippenberger S Bereiter-Hahn J Integration of Langerhans-like cells into a human skin equivalent. Arch Dermatol Res (2010) 303:135–9 10.1007/s00403-010-1092-x 21069532

[59] van Belkum AA Staphylococcal colonization and infection: homeostasis versus disbalance of human (innate) immunity and bacterial virulence. Curr Opin Infect Dis (2006) 19:339–44 10.1097/01.qco.0000235159.40184.61 16804380

[60] Quinn GA Tarwater PM Cole AM Subversion of interleukin-1-mediated host defence by a nasal carrier strain of *Staphylococcus aureus*. Immunology (2009) 128:e222–9 10.1111/j.1365-2567.2008.02952.x 19740308 PMC2753939

